# Embalment

**Published:** 1857-08

**Authors:** 


*Embalment.*—A writer in the New Orleans Medical Journal, Dr. W. T. Cox, gives an interesting article on the subject of embalming the dead. We are in error when we speak of this as one of the lost arts, or as one laid aside from a change in the customs of nations. The pomp and ceremony of modern burial often require the preservation of the remains of dead humanity, and in many of the nations of Europe the process is as common as it was of old on the banks of the sacred Nile; and much more perfect. Egyptian embalment was but a process of desiccation. The air of the valley of the Nile so rapidly hastens decomposition that, without some artificial process, the bodies of the departed could scarcely be preserved for a few hours. As soon as the breath had left the body it was borne to the sand ridges of the desert which skirts the valley, and there deposited in the nitrous sands. The scorching wind of Edom blew over it, its moisture rapidly disappeared, and it was soon brought down again to some temple of Isis, to be dipped in bitumen, and rolled in the linen bandages which remain to this day. "Forty days," says Holy Writ, speaking of the mourning of Joseph for his father Jacob, "And forty days were fulfilled for him; for so are fulfilled the days of those which are embalmed."

But the days of Egyptian greatness, and with them the reverence for the dead, are gone. Other and less revolting means of preservation are resorted to by the moderns. Among them the method of M. Gunnal, of which so much was said a few years since, is still prominent. It consists of an injection of a solution of alum and common salt, although it is charged that M. G. did not rely upon this alone, but added arsenite of soda largely to his preparation. Corrosive sublimate, or corrosive sublimate and creosote, have for a long time been successfully used. For the preservation of dead bodies for dissection, the medical colleges of Philadelphia rely mostly on chloride of zinc in solution, but it, like all other mineral injections, has many and important objections. In all ordinary cases, our personal experience teaches us that where subjects are to be preserved in the wet state, common salt is an adequate antiseptic. We have given the most thorough trial to this, by injecting, first, with a solution of nitrate of potassa; and then packing the subject in a brine, not so strong as to deposit any free salt. But a more usual and convenient method, and one by which a subject may be kept fit for dissection for two or three years, and for aught we know much longer, is to inject the femoral artery with alcohol containing a little creosote and corrosive sublimate, and then pack in sawdust, saturated with alcohol. In a large tank provided for this purpose, we have known six or eight subjects to lie for months in perfect preservation. One thing is remarkable, and that is that there are no means by which drowned subjects can be saved from rapid decomposition.

But that which is now most desirable for the art of embalming, is some process by which the body may be preserved in a dry state for the purposes of prolonged periods before interment; or permanently, in order that the decomposition of bodies in city cemeteries may not poison the air around. The use of poisonous salts is objectionable, both for reasons of health and because they would interfere in subsequent judicial investigations in cases of poisoning. The patent metallic burial case would aid very much in any such process, and we are inclined to believe that a body injected with creosote and alcohol, and subsequently placed in a metallic case, would last indefinitely. But this method is too expensive for general use, and it yet remains for those interested in the prosecution of this subject, to discover some means, by which the process of decay may be interrupted, or modified, so that the decomposition of a crowded church-yard may not sow death among the population around it.

